# The Potential of Induced Pluripotent Stem Cells to Test Gene Therapy Approaches for Neuromuscular and Motor Neuron Disorders

**DOI:** 10.3389/fcell.2021.662837

**Published:** 2021-04-13

**Authors:** Marisa Cappella, Sahar Elouej, Maria Grazia Biferi

**Affiliations:** Sorbonne University, INSERM, Institute of Myology, Center of Research in Myology, Paris, France

**Keywords:** IPSCs, gene therapy, AAV, NMD, MND, ASOs

## Abstract

The reprogramming of somatic cells into induced pluripotent stem cells (iPSCs) represents a major advance for the development of human disease models. The emerging of this technique fostered the concept of “disease in a dish,” which consists into the generation of patient-specific models *in vitro*. Currently, iPSCs are used to study pathological molecular mechanisms caused by genetic mutations and they are considered a reliable model for high-throughput drug screenings. Importantly, precision-medicine approaches to treat monogenic disorders exploit iPSCs potential for the selection and validation of lead candidates. For example, antisense oligonucleotides (ASOs) were tested with promising results in myoblasts or motor neurons differentiated from iPSCs of patients affected by either Duchenne muscular dystrophy or Amyotrophic lateral sclerosis. However, the use of iPSCs needs additional optimization to ensure translational success of the innovative strategies based on gene delivery through adeno associated viral vectors (AAV) for these diseases. Indeed, to establish an efficient transduction of iPSCs with AAV, several aspects should be optimized, including viral vector serotype, viral concentration and timing of transduction. This review will outline the use of iPSCs as a model for the development and testing of gene therapies for neuromuscular and motor neuron disorders. It will then discuss the advantages for the use of this versatile tool for gene therapy, along with the challenges associated with the viral vector transduction of iPSCs.

## Introduction: iPSCs, an Invaluable Resource for Disease Modeling

The development of human induced pluripotent stem cells (iPSCs) (Takahashi et al., 2007) provided unprecedented opportunities to decipher pathophysiological mechanisms of diseases and to test therapeutic approaches in conditions that better translate to humans. This technology allows to obtain an unlimited number of cells from one patient thus representing an ideal model to study *in vitro* disease’s developmental stages, onset and progression in specific human cells (Park et al., 2008a).

iPSCs are capable of indefinite self-renewal and can differentiate into any cell type under appropriate culture conditions (Takahashi et al., 2007; Yu et al., 2007). iPSCs are generated by reprogramming primary somatic cells, such as dermal fibroblasts or blood cells, using ectopic expression of selected embryonic transcription factors (e.g., Oct4, Sox2, Klf4, and c-Myc) (Takahashi et al., 2007). Over the years, several techniques have been refined to deliver the reprogramming cocktail for iPSCs generation. The first pioneering studies on iPSCs used integrating delivery systems, through retroviral or lentiviral vectors (Takahashi et al., 2007; Yu et al., 2007; Park et al., 2008b). To avoid any incorporation of the foreign genetic material and induction of genomic alterations (Nakagawa et al., 2008; Shao and Wu, 2010), novel delivery systems have been introduced, based on non-integrating vectors (such as the Sendai virus or episomal vectors), self-excising vectors (i.e., Cre-Lox, PiggyBac transposon), and non-viral vectors (i.e., combination of signaling molecules, small bioactive molecules, microRNAs, and other chemicals) (Liu et al., 2020). Interestingly, the delivery of synthetic mRNA expressing the reprogramming factors, was also exploited for the safe generation of iPSCs (Warren et al., 2010). It was also used for iPSCs differentiation (Warren et al., 2012; Mandal and Rossi, 2013; Yoshioka et al., 2013; Goparaju et al., 2017). This technology provides high *in vitro* transfection efficiency of complex mixtures, with transient expression and absence of genomic integration (Sahin et al., 2014).

iPSCs have the ability to retain the genetic mutation carried by the donor patient together with its genomic background, overcoming the limitations presented by the animal models and leading to a new era of disease modeling and clinical applications (Shi et al., 2017). Moreover, unlike the other unlimited sources of self-renewing cells, the embryonic stem cells (ESCs), which can only be obtained from early-stage blastocysts (4–5 days post fertilization), the iPSCs can be generated from adult patients, eliminating the ethical issues related to the generation of ESCs and leading to the opportunity for studying different stages of the disorders (Romano, 2008; Romito and Cobellis, 2016).

However, genetic background heterogeneity, lack of proper controls, as well as technical challenges in handling and standardizing the culture methods (Doss and Sachinidis, 2019; Volpato and Webber, 2020), contribute to the variability observed in the use of iPSCs as disease model (Hoekstra et al., 2017; Karagiannis et al., 2018; Volpato and Webber, 2020). To deal with genetic background influence on the expression of disease phenotype it is now possible to generate isogenic cell lines, introducing or repairing putative causative mutations through the clustered regularly interspaced short palindromic repeats (CRISPR)/Cas9-mediated genomic editing technologies (Ben Jehuda et al., 2018). The use of such controls, when possible, reduces the observed variation in cellular phenotypes caused by the genomic milieu (Soldner and Jaenisch, 2012).

Thanks to the mentioned superior features, iPSCs were exploited to generate *in vitro* models of severe diseases affecting the neuromuscular system and/or the central nervous system, such as neuromuscular and motor neuron disorders (NMD and MND, respectively). While genetic corrected iPSCs are investigated in the complex field of cell replacement therapies, in which modified cells are reintroduced into patients (Tedesco et al., 2012; Barthélémy and Wein, 2018; Abdul Wahid et al., 2019), the iPSCs platform has already allowed the identification of drug candidates for some of these complex disorders (Ortiz-Vitali and Darabi, 2019; Pasteuning-Vuhman et al., 2020). Recently, the combination of iPSCs and gene targeting approaches is changing the face of modern medicine. In this review, we will thus briefly discuss the successes in the identification of drug candidates for NMD and MND and then we will focus on the efforts toward the validation of gene therapy approaches in iPSCs for muscular dystrophies, amyotrophic lateral sclerosis (ALS) and spinal muscular atrophy (SMA). Table 1 summarizes the research efforts in this direction mentioned in this review.

**TABLE 1:** Summary of the major findings of the cited articles in which iPSCs were used for therapeutic tests of neuromuscular and motor neuron disorders.

**Therapeutic strategy**	**Differentiated cell model**	**Disease**	**Gene (Mutation)**	**Main results**	**References**
Small drugs	Myoblasts	DMD	*DMD* (c.457C > T)	Drug screening identified ginsenoside Rd and fenofibrate to enhance myogenic fusion of DMD iPSC-derived myoblasts.	Sun et al., 2020
	Motor neurons	Sporadic and familial ALS	*FUS* (p.H517D), *TDP-43* (p. M337V; p. Q343R) and SOD1 (p. H46R; p. H43R)	Drug screening and evaluation of multiple-phenotype rescue identified ROPI as a potential therapeutic candidate.	Fujimori et al., 2018
	Motor neurons	Sporadic ALS and familial ALS	*SOD1* (p.A4V)	Retigabine was identified as a drug that suppresses the hyperexcitability of ALS iPSC-derived MNs based on electrophysiological analysis.	Wainger et al., 2014
	Motor neurons	Sporadic and familial ALS	*SOD1* (p.L144FVX)	Drug screening identified bosutinib, a Src/c-Abl inhibitor that promotes autophagy and rescues ALS MN degeneration by inhibiting misfolded SOD1 aggregation and suppressing cell death in familial and sporadic ALS cases.	Imamura et al., 2017
	Motor neurons	SMA	*SMN1*	Drug screening identified a novel HDAC inhibitor that increases SMN2 mRNA levels.	Lai et al., 2017
	Motor neurons	SMA type I and II	*SMN1*	Test of RG7800 (first generation of risdiplam), a splice switching drug which increased SMN protein levels.	Ratni et al., 2016
	Motor neurons	SMA type I	*SMN1*	Test of risdiplam (FDA approved for SMA treatment), a splice switching drug which increased SMN levels.	Ratni et al., 2018
	Motor neurons	SMA type II	*SMN1*	Test of TEC-1, a splice switching drug which increased SMN levels.	Ando et al., 2020
ASOs	Cardiomyocytes	DMD	*DMD* (Exons 48–50 deletion; Exons 47–50 deletion; c.3217G > T; 4918-4919delTG; c.7437G > A)	ASO-mediated exon-skipping on exon 51 restored dystrophin to nearly 30% of the normal level.	Dick et al., 2013
	Skeletal muscle cells	DMD	*DMD* (Exon 44 deletion; Exons 46–47 deletion)	ASOs-mediated exon-skipping of exon 45 restored dystrophin protein expression and reduced calcium overflow.	Shoji et al., 2015
	Skeletal muscle cells	DM1	*DMPK1* (CTG repeats)	ASOs abolished RNA foci and rescued mis-splicing.	Mondragon-Gonzalez and Perlingeiro, 2018
	Motor neurons	ALS	*C9ORF72* repeat expansion	ASOs targeting the C9ORF72 transcript suppressed RNA foci formation and reversed gene expression alterations.	Sareen et al., 2013
	Mixed neuron-glia cultures	ALS	*C9ORF72* repeat expansion	ASOs targeting C9ORF72 rescues glutamate cytotoxicity and reversed disease-specific transcriptional changes.	Donnelly et al., 2013
	Motor neurons	ALS	*C9ORF72* repeat expansion	ASOs targeting C9ORF72 decreased intracellular and extracellular poly(GP) proteins.	Giorgio et al., 2019
	Motor neurons	ALS	*C9ORF72* repeat expansion	ASOs knocking down Ataxin-2, suppressed nucleocytoplasmic transport defects as well as neurodegeneration.	Zhang et al., 2018
	Motor neurons	ALS	*SOD1*	ASOs designed to reduce the synthesis of SOD1 increased survival of ALS iPSC-derived MNs and reduction of the misfolded SOD1 and the apoptotic markers expression.	Nizzardo et al., 2016
	SMA-iPSCs	SMA	*SMN1*	MOs targeting SMN2 to significantly increase SMN production.	Ramirez et al., 2018
	Motor neurons	SMA	*SMN1*	A new ASO variant resulted in a significant improvement of full-length SMN expression by correcting the aberrant splicing of SMN2 at the pre-mRNA level.	Osman et al., 2016
	Motor neurons	SMA	*SMN1*	Three molecular strategies: ASOs, exon-specific U1 small nuclear RNA and Transcription Activator-Like Effector-Transcription Factor increased SMN protein and rescued neuropathological features of SMA.	Nizzardo et al., 2015
AAV vectors	iPSCs	–	–	AAV3 vectors were used to introduce genetic modifications in iPSCs.	Mitsui et al., 2009
	iPSCs	–	–	AAV2 was used for gene targeting in iPSCs.	Khan et al., 2010
	iPSCs	–	–	Creation of a novel variant of AAV (AAV1.9) with a threefold higher gene delivery efficiency than AAV2.	Asuri et al., 2012
	Cardiomyocytes	–	–	AAV vectors preferentially transduced differentiated cells and identified serotypes 2 and 6 as the best suited for cardiomyocyte-iPSCs transduction.	Rapti et al., 2015
	Neuronal and glial cells	ALS	*C9ORF72* repeat expansion	AAV5 efficiently transduced 90% of the iPSCs-derived neuronal and glial cells, reducing the total expression of C9ORF72.	Martier et al., 2019a
	Retinal pigment epithelium and cortical neurons	*–*	*–*	The analysis of transduction efficiency using 11 AAV vectors (serotypes 1–9, 7m8, and 8b) showed substantial expression differences according to cell Type, MOIs and transduction time.	Duong et al., 2019
	Mature frontal brain-like neurons, Dopaminergic neurons, astrocytes, and motor neurons	ALS	*C9ORF72* repeat expansion	AAV5-miRNAs efficiently transduce different iPSC-derived cells reducing the amount of C9ORF72 transcripts.	Martier et al., 2019b
	iPSCs	*–*	*–*	A Testing Kit containing 30 AAV vectors was tested and showed that bioengineered vectors, such as AAV 7m8, AAV LK03, and AAV DJ were efficient in iPSCs transduction.	Westhaus et al., 2020
	iPSC-derived cerebral organoids and neural cells	*–*	*–*	AAV5 showed a higher transduction in organoids and neural cells when compared to AAV9.	Depla et al., 2020

**AAV, adeno associated viral vectors; ALS, amyotrophic lateral sclerosis; ASOs, antisense oligonucleotides; C9ORF72, chromosome 9 open reading frame 72; DM1, myotonic dystrophy 1; DMD, duchenne muscular dystrophy; DMD, Dystrophin; DMPK, dystrophia myotonica protein kinase; FDA, Food and Drug Administration; FUS, Fused in sarcoma; HDAC, histone deacetylase; iPSCs, induced pluripotent stem cells; miRNAs, microRNAs; MOI, multiplicity of infection; MNs, Motor neurons; ROPI, ropinirole; SMA, spinal muscular atrophy; SMN1, Survival of motor neuron 1; SOD1, Superoxide dismutase1; TDP-43, TAR-DNA-Binding Protein 43.**

## Drug Screening for Neuromuscular and Motor Neuron Disorders in a Dish, From Research Efforts to Clinical Application

iPSCs are widely exploited in high-throughput drug screenings for genetic disorders. Thus far, the introduction of iPSCs into the drug development pipeline has allowed (i) physiologically improved modeling of disease-relevant phenotypes, (ii) a greater patient stratification, and (iii) discrimination between drug responders and non-responders (Pasteuning-Vuhman et al., 2020). In perspective, this will have an impact on the current limitations of the conventional drug discovery process and consequently improve the success of therapeutic target identification and clinical trial outcomes (Hosoya and Czysz, 2016).

Following their discovery, multiple research efforts focused on the generation of iPSCs for NMD and MND. As example, in 2008 Park and collaborators, established the first iPSCs line from skin fibroblasts from a patient affected by Duchenne muscular dystrophy (DMD), a fatal genetic disorder caused by mutations in the *dystrophin* (*DMD)* gene and characterized by progressive muscle wasting (Koenig et al., 1987; Park et al., 2008a; Gao and McNally, 2015). Since then, additional DMD-iPSC lines have been reported by other groups and several differentiation protocols were tested to refine the optimal methods for skeletal muscle and cardiac cell differentiation (reviewed by Danisovic et al., 2018; Piga et al., 2019). These attempts overcame some of the limitations of the commonly used human models of DMD, such as myoblasts obtained from patient biopsies, which are limited in number and phenotypically diverse (Blau et al., 1983; Renault et al., 2000; Sun et al., 2020). In contrast, patient-derived iPSCs allow the generation of large amount of mature skeletal muscle cells (Chal et al., 2016; Caputo et al., 2020) or cardiomyocytes—recapitulating the cardiomyopathy of dystrophic patients (Hashimoto et al., 2016), and can mimic different stages of the disorder (Xia et al., 2018). iPSCs were also converted to neuronal cells to study the impact on the central nervous system in NMD. For example, neuron-iPSCs were generated from patients affected by myotonic dystrophy 1 (DM1) (Du et al., 2013; Xia et al., 2013; Ueki et al., 2017), caused by an expansion of the CTG trinucleotide repeats in the 3′ untranslated region of the *dystrophia myotonica protein kinase* (*DMPK*) gene (Brook et al., 1992). Altogether these studies highlight the versatility of iPSCs as model for the thorough study of gene mutations in the main affected tissues (i.e., skeletal and cardiac muscle for DMD) but also in other relevant cell types (such as neurons in DM1), which contribute to the disease manifestations. Furthermore, iPSCs are being exploited for the development of therapies for muscular dystrophies which is usually carried out in mouse models unable to fully recapitulate all the human disease features (Wells, 2018; Ortiz-Vitali and Darabi, 2019; van Putten et al., 2020). Recently, Sun and colleagues developed a platform based on DMD-iPSC–derived myoblasts for drug screening and among 1524 compounds analyzed, they identified 2 promising small molecules with *in vivo* efficacy (Sun et al., 2020). Further efforts in this direction will likely improve the search for reliable drug candidates and eventually increase the success rate in clinical trials for these severe disorders.

While animal models remain the preferred choice also for modeling and drug testing for MND (Picher-Martel et al., 2016; Dawson et al., 2018; Giorgio et al., 2019), the large genetic variability of these disorders set the ground for the wide use of patient-derived cells. Since 2008, when Eggan’s group (Dimos et al., 2008) used for the first time iPSCs to produce patient-specific motor neurons and glia from skin cells of an 82-year-old female patient diagnosed with ALS—the most common adult onset MND—several groups have designed and validated protocols for spinal motor neurons (MN) (Son et al., 2011; Amoroso et al., 2013; Demestre et al., 2015; Maury et al., 2015; Toli et al., 2015; Sances et al., 2016; Fujimori et al., 2018) and astrocyte differentiation (Madill et al., 2017; Birger et al., 2019; Zhao et al., 2020). The studies performed in ALS-iPSCs with different genetic mutations, facilitated the identification of common pathological features to the various disease forms, such as endoplasmic reticulum stress (Kiskinis et al., 2014; Dafinca et al., 2016), mitochondrial abnormalities (Dafinca et al., 2020; Hor et al., 2020), and impaired excitability (Wainger et al., 2014), but also characteristics related to specific mutations, like protein aggregation or mislocalization (Liu et al., 2015).

Drug screenings using ALS-derived iPSCs additionally allowed the identification of three drugs that are currently explored as therapeutic options in clinical trials.

- The first one, ROPI, a dopamine receptor agonist, was identified from a panel of 1232 Food and Drug Administration (FDA)-approved drugs in a drug screening analysis conducted at Keio University, which examined *Fused in sarcoma* (*FUS*)- and *TAR-DNA-Binding Protein 43* (*TDP-43*)-ALS iPSC-derived MN for suppression of ALS-related phenotypes *in vitro*, such as mislocalization of FUS/TDP43, stress granule formation, MN death/damage, and neurite retraction (Fujimori et al., 2018). This drug is now tested in the ROPALS trial (UMIN000034954 and JMA-IIA00397) as continuation of the Phase I/IIa clinical trial (Morimoto et al., 2019).

- Retigabine (known as an antiepileptic) was identified as a potential suppressor of the hyperexcitability of ALS iPSC-derived MNs based on electrophysiological analysis (Wainger et al., 2014). It is a voltage-gated potassium channel activator (Kv7) able to both block hyperexcitability and improve MN survival *in vitro* when tested in ALS cases carrying the most common genetic mutations (Wainger et al., 2014). A Phase II Pharmacodynamic Trial of Ezogabine (Retigabine) on neuronal excitability in ALS (NCT02450552) was conducted from 2015 to 2019 showing a decrease of cortical and spinal MN excitability in participants with ALS. These data suggest that such neurophysiological metrics may be used as pharmacodynamic biomarkers in multisite clinical trials (Wainger et al., 2020).

- The third drug is Bosutinib, a proto-oncogene non-receptor protein tyrosine kinase (Src/c-Abl) inhibitor that promoted autophagy and rescued degeneration in iPSC-derived MN, inhibiting misfolded Superoxide Dismutase 1 (SOD1) aggregation and suppressing cell death in genetic and sporadic ALS (Imamura et al., 2017). A new Phase I clinical trial of the drug bosutinib for ALS (UMIN000036295) was initiated in Japan in March 2019.

These examples of drug discovery in iPSCs and their ongoing translation to patients affected by a yet uncurable disease, indicate that this could be a valid paradigm for clinical success in similar diseases, such as SMA. SMA is a MND caused by homozygous mutations in the survival of motor neuron gene (*SMN1*) leading to infant mortality and motor disabilities in young and adult patients (Lefebvre et al., 1995; Verhaart et al., 2017; Smeriglio et al., 2020). This gene has a paralog called *SMN2* that is nearly identical to *SMN1*, with few nucleotide differences, which result in the exclusion of exon 7 and 90% production of a truncated non-functional survival of motor neuron (SMN) protein (Lefebvre et al., 1995). Several therapeutic strategies have been tested to restore SMN expression (Wirth, 2021). Histone deacetylase (HDAC) inhibitors were tested to induce transcriptional activation of *SMN2* and consequent increased production of full length SMN, with successful outcomes in proof-of-concept studies and failure in clinical trials. With the aim to identify compounds with higher efficacy and specificity, Lai and colleagues performed a drug screening in neuron-iPSCs from SMA patients. This study identified novel HDAC inhibitors with therapeutic potential that could be further explored for SMA treatment (Lai et al., 2017). Interestingly, neuron-iPSC from SMA patients were also used to test the efficacy of the recent FDA approved small molecule Evrysdi^TM^ (risdiplam) (Ratni et al., 2016; Ratni et al., 2018; Dhillon, 2020), which forces the inclusion of exon 7 and thus restore SMN protein levels (Poirier et al., 2018). Moreover, the drug called TEC-1 (2-(4,6-dimethylpyrazolo[1,5-a]pyrazin-2-yl)-6-(4-methylpiperazin-1-yl)quinazolin-4(3H)-one) another *SMN2* splicing modulator, was recently identified in a screening on SMA patient-derived fibroblasts. The drug’s effects were then confirmed in SMA-MN-iPSCs (Ando et al., 2020).

**FIGURE 1:**
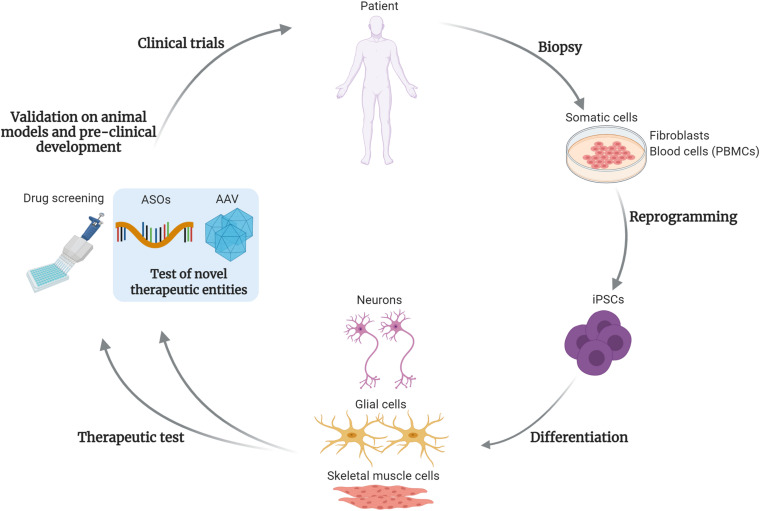
Test and development of gene targeting approaches using iPSCs. This drawing summarizes the steps of development for drugs and gene therapy approaches, using induced pluripotent stem cells (iPSCs). Somatic cells, such as fibroblasts or blood cells (peripheral blood mononuclear cells, PBMCs) are obtained from patient’s biopsies. After reprogramming, the patient-derived iPSCs can be differentiated into disease-relevant cell types, such as skeletal muscle cells, neural or glial cells for neuromuscular or motor neuron disorders. These cells are then subjected to the classical high-throughput drug screening and in perspective will be used to test novel therapeutic entities, based on gene targeting approaches. As example, antisense oligonucleotides (ASOs) or adeno-associated viral vectors (AAV)-based strategies. After validations in animal models and the pre-clinical development process, these novel therapies could enter into clinical trials for patients affected by rare disorders. The use of iPSCs and gene targeting strategies will likely foster the development of personalized medicine approaches. Created with BioRender.com.

As suggested by the reported examples, the combination of iPSCs modeling, together with high-throughput drug screening followed by animal tests will likely ensure the identification of effective and safe therapeutic candidates. How this pipeline can be adapted to the development and tests for precision medicine approaches, such as gene therapy, will be discussed in the following paragraphs and is exemplified in Figure 1.

## iPSCs for Gene Therapy, a Focus on Antisense Oligonucleotides

Gene targeting approaches are based on the direct correction of the genetic defects (Wang and Gao, 2014; Cappella et al., 2019). For example, antisense oligonucleotides (ASOs) widely tested in pre-clinical and clinical settings, have been approved for SMA (Spinraza^®^) (Aartsma-Rus, 2017) and DMD (i.e., Exondys 51^®^) (Stein, 2016) patients, encouraging their use for the treatment of other monogenic disorders.

ASOs are synthetic single-stranded strings of nucleic acids that bind to RNA through standard Watson–Crick base pairing. After binding to the targeted RNA, the antisense drug can modulate the function of the targeted RNA by several mechanisms (Bennett and Swayze, 2010; Crooke et al., 2018), depending on the chemical modifications and the binding position on the target RNA (Wurster and Ludolph, 2018; Talbot and Wood, 2019; Ochoa and Milam, 2020). Briefly, ASOs can promote degradation of the targeted RNA, by mimicking DNA-RNA pairing and activating endogenous nucleases (i.e., RNase H1), or can modulate the processing of the RNA molecule, without inducing its degradation. This can be achieved through several mechanisms, such as by masking RNA splicing sites, as in the examples described below for DMD or SMA (Dick et al., 2013; Shoji et al., 2015; Osman et al., 2016; Ramirez et al., 2018). Other methods of action of ASOs have been previously reviewed (Bennett and Swayze, 2010; Crooke, 2017).

Several strategies, (Miller and Harris, 2016; Schoch and Miller, 2017), are currently investigated to increase ASOs stability, enhance binding affinity to the target RNA, improve tissue distribution and cellular uptake, while decreasing possible adverse effects (Bennett et al., 2017). Here we will focus on the use of iPSCs as model for testing the efficacy of these gene targeting approaches in NMD and MND.

Due to the large size of the *DMD* gene (Koenig et al., 1987), the restoration of the full-length dystrophin protein is challenging (Gao and McNally, 2015; Duan, 2018). One of the most promising approaches for gene targeting in DMD, is the use of ASOs binding to the pre-mRNA of the *DMD* gene to restore its reading frame and consequently producing a truncated but yet functional protein.

The ASO-mediated exon-skipping efficacy on exon 51 was tested in cardiomyocytes derived from iPSCs with *DMD* mutations, restoring dystrophin to nearly 30% of the normal level (Dick et al., 2013). Another similar study tested an ASO forcing exon 45 skipping of the *DMD* gene in myotubes derived from iPSCs, thus restoring dystrophin expression but also reducing calcium overflow (Shoji et al., 2015). These studies indicate that iPSCs can be used as platforms for therapeutic selection of ASO, based on the gene correction and prevention of skeletal muscle phenotype in DMD. The new frontier for the treatment of DMD patients is the development of mutation-specific ASOs (Schneider and Aartsma-Rus, 2020) and the use of iPSCs will likely speed the path to success of those strategies through the selection of the patient-specific and most efficient candidates.

ASOs were also proven effective in differentiated myotubes from DM1-iPSCs. A repeat-directed ASO treatment abolished RNA foci accumulation and rescued mis-splicing (Mondragon-Gonzalez and Perlingeiro, 2018) *in vitro*. These discoveries indicate that once established the proper conversion and differentiation protocols, together with valid disease read-outs, the test of ASOs in iPSCs could be likely applied to a larger spectrum of muscular dystrophies and diseases.

Therapeutic ASOs are currently tested in clinical trials for ALS patients harboring the chromosome 9 open reading frame 72 (*C9ORF72*) mutations (NCT03626012), *SOD1* mutations (NCT03070119, NCT02623699) (recently reviewed by Cappella et al., 2021) or for sporadic ALS patients, with the Ataxin2-ASO (NCT04494256, Becker et al., 2017). Importantly, a splice switching ASO targeted to *SMN2* (Spinraza^®^) was approved for SMA patients in 2016.

To better characterize ASOs ability to rescue disease hallmarks, to dissect pathophysiological mechanisms and to test novel chemistries and molecular technologies, different research groups are studying ASOs in iPSCs for MND. For example, ASOs were proven effective in reducing the accumulation of sense RNA foci or toxic dipeptides in C9ORF72-iPSCs differentiated to neurons or MN (Donnelly et al., 2013; Sareen et al., 2013; Giorgio et al., 2019). More recently, Zhang et al. (2018) demonstrated that nucleocytoplasmic transport deficits and neurodegeneration were alleviated in C9ORF72-MN-iPSCs, after treatment with ASOs directed against the Ataxin 2, an RNA-binding protein. Nizzardo et al. (2016) treated ALS MN-iPSCs with ASOs designed to reduce the synthesis of human SOD1 and observed an increased survival and reduced expression of apoptotic markers in treated cells.

In SMA, iPSCs were used to test novel ASO sequences for their improved capacity of producing the full length SMN protein from splicing modulation of *SMN*2 and exon 7 inclusion (Osman et al., 2016; Ramirez et al., 2018). They were also used to test novel molecular strategies to restore SMN expression and correct neuropathological feature, namely an U1 small nuclear RNA-mediated splice switching approach and SMN transcription activation, *via* the Transcription Activator-Like Effector-Transcription Factor (TALE-TF) (Nizzardo et al., 2015). This report suggests that iPSCs could serve for the side-by-side comparison of different gene targeting strategies for monogenic disorders.

## iPSCs as a Model for AAV-Based Gene Therapy Testing

The use of adeno-associated viral vectors (AAV) for gene therapy of rare disorders recently became a clinical reality. The approval of Zolgensma^®^ (an AAV-mediated therapy) for the treatment of the most severe form of SMA, endorses the development of similar approaches for NMD and MND. Indeed, several pre-clinical studies report successes of these approaches in disease models (Biferi et al., 2017; Cappella et al., 2019; Crudele and Chamberlain, 2019) and their use in clinical trials (Bowles et al., 2012; Mendell et al., 2015; Mueller et al., 2020).

Some of the challenges associated to the translation of AAV-based therapies from animal models to patients, are linked to (i) the selection of the best AAV serotype for efficient transgene expression, (ii) cell/tissue specificity, as well as (iii) production of high vector titers, and (iv) reduction of immunoreactivity (Colella et al., 2017; Naso et al., 2017). To date, hundreds of natural AAV serotypes, variants and bio-engineered versions have been described (Hester et al., 2009; Choudhury et al., 2016; Deverman et al., 2016; Chan et al., 2017; Hanlon et al., 2019). Beside serotypes, research efforts are also focusing on the combination of the best serotype with the therapeutic and regulatory sequences—such as promoters or enhancers (Colella et al., 2018; Besse et al., 2020; Nieuwenhuis et al., 2020), for efficient, safe and specific transgene expressions (Guilbaud et al., 2019; Hanlon et al., 2019). This will likely contribute to expedite the translational path from bench to clinic. In this context, iPSCs can be used to select the vector with best transduction properties for a specific cell type and/or to test the therapeutic sequences (recombinant transgene, oligonucleotides, antibodies, etc.). These techniques will be further refined to design patient-specific approaches. In perspective, when a therapeutic candidate will be established, iPSCs could be further used for analytical tests of approved gene therapies, such as potency assays.

AAV vectors were initially tested for genetic manipulation of ESCs or iPSCs *in vitro*, using natural human-derived AAV serotypes (from 1 to 9). After some unsuccessful attempts (Smith-Arica et al., 2003; Jang et al., 2011), some reports showed that natural AAV vector serotypes, such as AAV 2 and 3, were able to target iPSCs, although with limited efficacy (Mitsui et al., 2009; Khan et al., 2010). Through direct evolution, Asuri et al. (2012), derived a novel variant of AAV (AAV1.9) with a threefold higher gene delivery efficiency than AAV2 in iPSCs. These pioneer studies suggested that AAV vectors could be also used for stem cell correction and consequently studies of biological mechanisms *in vitro* and eventually for therapeutic purposes in cell therapy approaches.

Several studies reported method for AAV-mediated delivery of differentiated iPSCs. For example, Rapti et al. (2015) compared the transduction efficiency of different AAV (serotypes 1, 2, 6, and 9) in cardiomyocyte-iPSCs. Interestingly, they noticed that AAV vectors preferentially transduced differentiated cells and identified in serotypes 2 and 6 the best suited for cardiomyocyte-iPSCs transduction.

For modeling and therapeutic testing of central nervous system cells, AAV serotype 5 expressing the green fluorescent protein (GFP), was proven efficient in iPSCs-derived neuronal and glial cells, resulting in up to 90% of transduction (Martier et al., 2019a). Moreover, Duong et al. examined the level of AAV-GFP expression following the transduction of 11 AAV vectors in iPSCs differentiated into retinal pigment epithelium and cortical neurons (Duong et al., 2019). GFP-expressing cells were examined and compared across doses, time and cell type. They reported that retinal pigmented epithelium had the highest AAV-mediated GFP expression compared to cortical neurons-iPSCs and that AAV7m8 and AAV6 were the best performing, across vector concentrations and cell types. This study suggested that in addition to vector tropisms, cell type significantly affects transgene expression (Duong et al., 2019).

Overall, following optimizations, AAV vectors can be used to efficiently transduce patient-derived cells converted to neural or glial cells, likely facilitating studies for neurological diseases. Indeed, Martier and colleagues investigated the feasibility of a miRNA-based gene therapy to obtain long-term silencing of the repeat-containing transcripts of *C9ORF72*. Four AAV5 carrying miR candidates were tested in neuron-iPSC, resulting in sufficient transduction and expression of therapeutically relevant levels of the corresponding mature miRNA (Martier et al., 2019b). Two of the tested candidates were then proven efficient in reducing RNA foci accumulation in some brain regions of a disease mouse model (Martier et al., 2019a).

Novel methods are currently developed to select AAV for their fitness *in vitro*. For example, the group of Lisowski developed an AAV Testing Kit, as novel high-throughput approach based on next-generation sequencing, to study the performance of 30 published AAV variants *in vitro*, *in vivo*, and *ex vivo*. They tested AAV variants in primary cells, immortalized cell lines and iPSCs, showing that iPSCs were most efficiently transduced with bioengineered vectors, such as AAV 7m8, AAV LK03, and AAV DJ (Westhaus et al., 2020). This suggests that further methods for AAV optimization are necessary and will likely improve AAV transduction properties *in vitro* and *in vivo*.

Transduction properties of AAV serotypes in the human context have been recently tested in 3D structure iPSC-derived cerebral organoids. The transduction properties of two commonly used AAV serotypes (AAV5 and 9) were compared for transgene expression at the mRNA and protein levels, together with the presence of viral DNA. This study reported a higher transduction of the AAV5 compared to AAV9, in organoids and neural cells (Depla et al., 2020). This work set the ground for the use of iPSCs-derived human organoids as valid system for testing AAV properties and will be likely a valuable platform for holistic characterization of AAV properties *in vitro* and identification of the best therapeutic candidates.

## Discussion

Gene therapy treatments are revolutionizing the face of modern medicine opening treatment perspectives for patients affected by fatal conditions. Despite the growing success of these approaches, several aspects of gene therapy development need refinement and would benefit of the use of iPSCs. Indeed, together with their most known use, such as disease modeling for high-throughput drug screenings, they can be converted into a reliable platform for testing the novel therapeutic entities. Indeed, after the establishment of proper differentiation protocols and disease readouts, patient-derived models are being utilized to test gene targeting approaches. Here, we have summarized research efforts in testing drugs and gene therapy approaches in iPSCs from patient affected by neuromuscular and motor neuron diseases. We have presented some of the successes in candidate drug identification, such as risdiplam for the treatment of SMA and the research efforts in testing ASOs and AAV-mediated therapies. These studies set the ground for further developments, to select optimized therapeutic molecules and to identify powerful and safe AAV vectors.

In parallel to iPSCs development, research efforts are currently focused on the generation of even more advanced disease models. Indeed, despite iPSCs represent a reliable model for the understanding of pathological mechanisms and therapeutic development, they do not fully recapitulate the complexity of a tissue, with its architecture and interactions (Costamagna et al., 2019). In this direction, 3D culture methods are being implemented for NMD and MND, for example with the generation of artificial skeletal muscle for DMD (Maffioletti et al., 2018) or spinal cord organoids for SMA, which were used for drug test (Hor et al., 2018). Interestingly, the group of Pasça, has recently reported the generation of iPSC-derived 3D culture, in which cerebral cortex or hindbrain/spinal cord organoids were assembled with skeletal muscle spheroids (Andersen et al., 2020). These so-called 3D cortico-motor assembloids hold promise for the development of effective therapeutics for NMD and MND.

In conclusion, the advances in novel technologies, such as production of mature organoids, will endorse the development of efficient personalized medicine approaches.

## Author Contributions

MC and SE: writing of the manuscript draft. MB: conceptualization, writing, and review. All authors contributed to the article and approved the submitted version.

## Conflict of Interest

The authors declare that the research was conducted in the absence of any commercial or financial relationships that could be construed as a potential conflict of interest.
